# Rate of cardiac arrhythmias and silent brain lesions in experienced marathon runners: rationale, design and baseline data of the Berlin Beat of Running study

**DOI:** 10.1186/1471-2261-12-69

**Published:** 2012-08-31

**Authors:** Karl Georg Haeusler, Juliane Herm, Claudia Kunze, Matthias Krüll, Lars Brechtel, Jürgen Lock, Marc Hohenhaus, Peter U Heuschmann, Jochen B Fiebach, Wilhelm Haverkamp, Matthias Endres, Gerhard Jan Jungehulsing

**Affiliations:** 1 Department of Neurology, Charité Berlin Campus Benjamin Franklin, Hindenburgdamm 30, 12203, Berlin, Germany; 2 Center for Stroke Research University Medicine Berlin, Campus Benjamin Franklin, Hindenburgdamm 30, Berlin, 12203, Charité, Germany; 3 SMS Sports Medicine Berlin, Medical Institute of the BMW BERLIN-MARATHON, , Germany; 4 Berliner Akademie für Sportmedizin e.V, Berlin, Germany; 5 Department of Sports Medicine, Humboldt-University, Berlin, Germany; 6 Institute of Clinical Epidemiology and Biometry, Center for Clinical Studies and Comprehensive Heart Failure Center, University Hospital, Würzburg, Germany; 7 Department of Cardiology, Campus Vichow-Klinikum, Berlin, Charité, Germany; 8 Excellence Cluster NeuroCure, Charité - Universitätsmedizin Berlin, Berlin, Germany

**Keywords:** Marathon running, ECG-recording, Magnetic resonance imaging, Blood sampling, Cardiac arrhythmia

## Abstract

**Background:**

Regular exercise is beneficial for cardiovascular health but a recent meta-analysis indicated a relationship between extensive endurance sport and a higher risk of atrial fibrillation, an independent risk factor for stroke. However, data on the frequency of cardiac arrhythmias or (clinically silent) brain lesions during and after marathon running are missing.

**Methods/ Design:**

In the prospective observational “Berlin Beat of Running” study experienced endurance athletes underwent clinical examination (CE), 3 Tesla brain magnetic resonance imaging (MRI), carotid ultrasound imaging (CUI) and serial blood sampling (BS) within 2-3 days prior (CE, MRI, CUI, BS), directly after (CE, BS) and within 2 days after (CE, MRI, BS) the 38^th^ BMW BERLIN-MARATHON 2011. All participants wore a portable electrocardiogram (ECG)-recorder throughout the 4 to 5 days baseline study period. Participants with pathological MRI findings after the marathon, troponin elevations or detected cardiac arrhythmias will be asked to undergo cardiac MRI to rule out structural abnormalities. A follow-up is scheduled after one year.

**Results:**

Here we report the baseline data of the enrolled 110 athletes aged 36-61 years. Their mean age was 48.8 ± 6.0 years, 24.5% were female, 8.2% had hypertension and 2.7% had hyperlipidaemia. Participants have attended a mean of 7.5 ± 6.6 marathon races within the last 5 years and a mean of 16 ± 36 marathon races in total. Their weekly running distance prior to the 38^th^ BMW BERLIN-MARATHON was 65 ± 17 km. Finally, 108 (98.2%) Berlin Beat-Study participants successfully completed the 38^th^ BMW BERLIN-MARATHON 2011.

**Discussion:**

Findings from the “Berlin Beats of Running” study will help to balance the benefits and risks of extensive endurance sport. ECG-recording during the marathon might contribute to identify athletes at risk for cardiovascular events. MRI results will give new insights into the link between physical stress and brain damage.

**Trial registration:**

clinicaltrials.gov NCT01428778

## Background

Regular physical exercise is becoming more and more popular in modern societies. Besides reflecting a healthy life style, endurance sport is beneficial for cardiovascular health [1,2], and lowers the risk of cancer [3] or mood disorders [4]. With regard to the increasing number of marathon runners in recent years, it remains an open question whether vigorous physical exercise, such as marathon running, might have harmful effects on heart and brain.

One might argue that sudden cardiac deaths occur rarely in marathon runners [5] despite of the fact that regular physical activity reduces the risk of coronary events during exercise [6]. Moreover, right ventricular dysfunction [7] and troponin elevations are common during marathon running, but these transient effects do not appear to cause cardiac ischemic injury [8,9]. However, a recent meta-analysis of mostly retrospective, small case-control series indicated that intense endurance sport increases the long-term risk of atrial fibrillation (AF) [10], while the underlying mechanisms and the impact of higher levels of physical exercise are poorly understood [11,12]. These findings raised concerns and afford further investigation because individuals with (even paroxysmal) AF have a four- to fivefold increased risk of ischemic stroke [13,14]. Moreover, clinically silent strokes frequently occur in AF-patients [15] and contribute to cognitive decline in the long-term [16].

The identification of endurance athletes at risk for cardiovascular events is challenging and despite of existing recommendations for medical screening in athletes [17,18], there is an ongoing debate about efficacy of routine screening and the impact of false-positive results [19]. Therefore, prospective studies are needed to characterize the preventive value of cardiac monitoring [20,21]. In particular, eligible data on the frequency of AF and other cardiac arrhythmias *during* endurance sport are missing so far [22]. Moreover, the frequency of (clinically silent) brain lesions during endurance sport is unknown. However, there are case reports on hyponatremic encephalopathy [23] and on non-significant changes of brain specific biomarkers [24], indicating potential brain damage during a marathon race.

To answer these questions, we initiated the prospective and observational “Berlin Beat of Running” study. By using a continuous ECG-monitoring we focus on the frequency of (clinically silent) cardiac arrhythmias in healthy endurance athletes before, during and after the 38^th^ BMW BERLIN-MARATHON 2011. By conducting MRI measurements at 3 Tesla (T) and serial laboratory tests immediately before and after the marathon race we hope to set new standards for further studies in endurance athletes.

## Methods / Design

### Study population and study design

The “Berlin Beat of Running” study is a prospective, observational, investigator-initiated study conducted by the Center for Stroke Research Berlin, Charité Berlin in cooperation with the Department of Cardiology, Charité (Campus Virchow-Klinikum), and the Medical Institute “SMS Sports Medicine Berlin” of the BMW BERLIN-MARATHON. The BMW BERLIN-MARATHON is one of the world’s largest marathons and more than 42.000 runners took part in 2011 (http://www.bmw-berlin-marathon.com). The “Berlin Beat of Running” study protocol is in accordance with the Helsinki declaration and was approved by the local Ethics Committee (EA4/042/11). Recruitment of athletes was supported by the SCC EVENTS GmbH (Sporting Club Charlottenburg, SCC), the organizer of the 38^th^ BMW BERLIN-MARATHON. Study entry and storage of data required subsequent consent by the participants. The inclusion and exclusion criteria are summarized in Table 1.

**Table 1:** The “Berlin Beat of Running” study: Inclusion and exclusion criteria

**Inclusion criteria**	
· Preregistered participant of the 38^th^ BMW BERLIN-MARATHON 2011	
· Age ≥ 35 and ≤ 60 years	
· Marathon history: at least 2 marathon runs within the last 5 years	
· Amount of physical activity: running for at least 40 km per week	
· Sinus rhythm on the day of enrolment	
**Exclusion criteria**	
· Known cardiac disease or cardiac arrhythmia	
· Known stroke or TIA, brain tumour or infectious disease affecting the brain	
· Contraindications for magnetic resonance imaging	
· Acute cerebral infarction or other clinical relevant pathological findings detected by brain magnetic resonance imaging before the marathon	
· Severe liver or kidney disease	
· Hyperthyroidism, pregnancy or lactation	

Baseline visit was done within three days before the 38^th^ BMW BERLIN-MARATHON. After informed consent, sociodemographics, the prevalence of cardiovascular risk factors, and the level of physical activity were assessed. Participants underwent assessment of vital parameters (heart rate and blood pressure), neurological examination (NIHSS score [25]) and venous blood sampling (complete blood cell count, ions, cardiac enzymes, renal function, neuron-specific enolase, endothelial markers, i.e. circulating endothelial microparticles, sICAM, Endothelin-1 and VEGF). The intima-media thickness was measured by using B-mode ultrasound (Esaote Mylab 25, ESAOTE Biomedica Deutschland GmbH, Köln, Germany) [26]. Brain MRI at 3 T was performed (see below). Finally, a portable ECG-recorder (CardioMem® CM 4000; GETEMED AG, Teltow, Germany; see below) was mounted by using five chest electrodes and ECG-recording was started. On marathon day, all remaining participants attended the study team within two hours before the start to ensure proper ECG-recording during the race. Within 30 minutes after the end of the marathon race vital parameters were assessed, and a second blood sampling (parameters as stated above) was done. ECG-recording was continued after the marathon race until the second brain MRI was done (within 20-58 hours after the end of the 38^th^ BMW BERLIN-MARATHON 2011). Afterwards the third blood sampling (parameters as stated above), assessment of vital parameters, neurological examination and Holter recording was done at the Charité.

All participants with detected atrial fibrillation, MRI-detected ischemic stroke or troponin elevation will undergo contrast-enhanced cardiac MRI. Participants with new brain lesions after the marathon will undergo additional brain MRI three months after the second brain MRI. Regular follow-up of all participants will take place 12 months after the marathon and will include an additional long-term ECG-recording. The final results of “Berlin Beat of Running” study are expected at the end of the year 2012.

### Aims and objectives

The aims of this “Berlin Beat of Running” study are: (1) to identify the frequency of cardiac arrhythmias in experienced marathon runners; (2) to become aware of impact factors associated with cardiac arrhythmias; (3) to analyse the frequency of (clinically silent) MRI-detected brain lesions before and immediately after a marathon race; (4) to correlate pathological findings of heart and brain to laboratory results; (5) to rule out myocardial scars in athletes with elevated troponin levels after the marathon; (6) to demonstrate feasibility of valid ECG-recording during enduring sports.

The primary hypothesis of the “Berlin Beat of Running” study is: (1) Cardiac arrhythmia and especially AF is frequently found in experienced marathon runners. Therefore, the primary outcome is the number of marathon runners with newly diagnosed cardiac arrhythmias. Secondary hypothesis are: (2) There are predictable risk factors associated with cardiac arrhythmias in marathon runners. (3) Marathon running is associated with (clinical silent) brain lesions, especially in those athletes with cardiac arrhythmias. The corresponding outcome parameter is the number of marathon runners with newly diagnosed brain lesions according to 3 T MRI after the marathon race. (4) Pathological laboratory findings are in part associated with cardiac arrhythmias and MRI findings of heart and brain. (5) Marathon runners with elevated troponin levels do not have MRI-detected myocardial scars suggestive for myocardial infarction. The corresponding outcome parameter will be assessed by using a contrast-enhanced cardiac MRI in participants with troponin elevation after the marathon race. Primary feasibility endpoint is the ability of the participants to complete the marathon despite of wearing the ECG-recorder.

### MRI analysis

Using a 3 T MR scanner (Magnetom Tim Trio; Siemens AG, Erlangen, Germany) the following sequences were done within 22-74 hours before as well as 20-58 hours after the marathon race: T2*-weighted imaging to screen for intracranial hemorrhage; diffusion-weighted imaging (DWI) to assess cerebral infarction and/or vasogenic oedema; Fluid-attenuated inverse recovery (FLAIR) to estimate microangiopathic lesions load and to investigate the age of recent lesions; time-of-flight MR-Angiography (TOF MRA) to detect vessel stenosis or occlusion [27]. Three month after the marathon a further measurement will be performed in those athletes with (clinically silent) brain lesions. Athletes with (clinically silent) brain lesions, cardiac arrhythmias or relevant troponin elevations will be asked to undergo cardiac 3 T MRI (Magnetom Tim Trio; Siemens AG, Erlangen, Germany) with a phased array receiver coil during breath-holds gated to the electrocardiogram (Body Matrix-coil #TATS; Siemens AG). Cine images of 3 long-axis as well as 14-18 short-axis views (slices of 4 mm) using a steady state free precession technique [28]. Eight minutes after i.v. administration of 10-12 ml Gadovist® (Bayer Schering Pharma AG, Berlin, Germany) at a concentration of 1 mmol/ml the above mentioned views will be repeated using a short inversion recovery sequence continuously adjusting the inversion time [29]. Blinded MRI reading will be done by a board certified (neuro-)radiologist (JBF).

### ECG-recording

The portable CardioMem® CM 4000 (GETEMED AG, Teltow, Germany) is a 5-lead, high-resolution digital Holter ECG-recorder, which performs continuous ECG-recording up to five days without changing the battery. Commercial electrodes (Blue Sensor SP; Ambu Inc, Glen Burnie, Maryland, USA) were placed in standard positions and fixed with additional tape. Acquired ECG-data were stored on the integrated memory card and transferred to a PC via the Hi-Speed USB interface. The analyses provided by the Holter system CardioDay® (GETEMED AG) includes heart rate, heart rate variability, ST-analysis, detection of atrial fibrillation as well as additional specialised tools such as heart rate turbulence, deceleration capacity and T-wave alternans (GE Healthcare, Milwaukee, WI, USA). ECG-reading and interpretation was performed off-line by an experienced physician.

### Sample size calculation and statistical analysis

The precision analysis is based on 100 ECG-monitored marathon runners. We hypothesize an odds ratio of 5.3 for atrial fibrillation in marathon runners compared to the normal population [10]. Assuming an AF-prevalence of 1% to 2% within the general population [30], this would correspond to an AF-prevalence between 5.1% and 9.8% in marathon runners. We intend to use the Wilson confidence interval as a basis, because we anticipate good properties regarding expected confidence interval length and coverage probability over a broad range for actual rates. First scenario: General AF-prevalence of 1% in the normal population: A sample size of 100 will provide a two-sided 95% confidence interval with a width equal to 0.091 with lower limit 0.022 and upper limit 0.113 when the sample proportion for marathon runners is 0.051. Second scenario: General AF-prevalence of 2% in the normal population: A sample size of 100 produces a two-sided 95% confidence interval with a width equal to 0.118 with lower limit 0.054 and upper limit 0.172 when the sample proportion for marathon runners is 0.098. Furthermore, we will report the proportion of (clinically silent) brain lesions within the group of marathon runners during the race. Due to the expected low number of events, it seems to be not feasible to compare the observed proportion for silent ischemic stroke statistically. Assuming a drop out rate of 10%, we included 110 marathon runners.

Descriptive statistics will be done regarding the outcome variables. For quantitative data, we will compute absolute and relative frequencies. In the case of continuous or quasi-continuous variables with nearly symmetric distribution, we will compute arithmetic mean, standard deviation, minimal and maximal values, otherwise median, quartiles, minimal and maximal values. Fisher’s exact test will be used to compare proportions for dichotomous outcomes between independent groups or to test independency of two dichotomous data within a population. Ordinal scaled outcomes will be compared by the t-test or the Mann-Whitney test, respectively, depending on normality or non-normality of distribution. The data presented here have been analyzed accordingly.

## Results

### Baseline characteristics of study participants

Overall, 110 athletes aged 36-61 years were enrolled (Table 2). Mean age was 48.8 ± 6.0 years, 24.5% (n = 27) were female, 8.2% (n = 9) had hypertension, and 2.7% (n = 3) had hyperlipidaemia. Before the 38^th^ BMW BERLIN-MARATHON 2011, the participants attended a mean of 7.5 ± 6.6 (range 2-50) marathon races within the last 5 years and a mean of 16.1 ± 35.8 (range 2-359) marathon races in total. Their average weekly running distance prior to the race was 64.7 ± 17.3 km (range 40-120 km). In addition, 45.5% (n = 50) of the enrolled participants regularly cycle and 18.2% (n = 20) regularly swim. Besides a lower body mass index and a lower systolic blood pressure in women, there were no gender-specific differences (Table 2).

**Table 2:** Baseline characteristics of the “Berlin Beat of Running” cohort (n = 110)

	**Total**	**Female**	**Male**	**p**
	**n = 110**	**n = 27**	**n = 83**	
Age; years; mean (SD)	48.8 (6.0)	48.8 (5.8)	48.8 (6.1)	0.939
Age ≥ 50 years; % (n)	40.0 (44)	40.7 (11)	39.8 (33)	0.928
Physical activity				
Marathon runs ≤ 5 years; mean (SD)	7.5 (6.6)	5.8 (3.3)	8.0 (7.3)	0.235
Marathon runs total; mean (SD)	16.0 (35.8)	10.0 (7.9)	18.0 (40.8)	0.247
Current weekly running; km; mean (SD)	64.8 (17.3)	60.2 (12.4)	66.1 (18.4)	0.207
Regular weekly running; km; mean (SD)	42.3 (13.6)	39.4 (11.9)	43.2 (14.1)	0.207
Systolic BP*; mmHg; mean (SD)	127.8 (12.9)	122.8 (9.0)	129.5 (13.6)	**0.016**
Diastolic BP*; mmHg; mean (SD)	83.7 (8.4)	81.7 (9.0)	84.4 (8.1)	0.327
Pulse; 1/min; mean (SD)	61.1 (8.2)	61.9 (8.2)	60.9 (8.2)	0.777
BMI^§^; kg/m^2^; mean (SD)	23.2 (2.2)	22.2 (2.3)	23.5 (2.0)	**0.004**
NIHSS^#^ score; mean (SD)	0 (0)	0 (0)	0 (0)	1.000
Comorbidities; % (n)				
Hypertension	8.2 (9)	0 (0)	10.8 (9)	0.074
Diabetes mellitus	0 (0)	0 (0)	0 (0)	1.000
Heart failure	0 (0)	0 (0)	0 (0)	1.000
Coronary artery disease	0 (0)	0 (0)	0 (0)	1.000
Hyperlipidaemia	2.7 (3)	0 (0)	3.6 (3)	0.317
Alcohol abuse; % (n)	0 (0)	0 (0)	0 (0)	1.000
Current smoking; % (n)	6.4 (7)	3.7 (1)	7.2 (6)	0.515

* Blood pressure; ^§^ Body Mass Index; ^#^National Institutes of Health Stroke Scale.

One participant had to be excluded due to brain MRI findings. Overall, 109 (99.1%) of all 110 participants took part and 108 (98.2%) participants finished the 38^th^ BMW BERLIN-MARATHON 2011 in 249 min ± 43 min in average. One participant quitted the marathon race due to muscular cramps after 34 kilometres but attended all regular study visits.

## Discussion

Regular physical activity improves the cardiovascular risk profile and lowers the overall mortality [11]. However, recent reports gave reasons to question the benefit of vigorous exercise, but there is little evidence from prospective studies so far [10,12]. Performing the “Berlin Beat of Running” study, we focus on safety aspects of marathon running by analyzing the frequency of (clinically silent) cardiac arrhythmias as well as of brain lesions in healthy endurance athletes running a marathon. This is the first approach in such a cohort using feasible long-time ECG-recording and high-resolution DWI at 3 T. As demonstrated before [31], high-resolution DWI is able to reveal about twice as many cerebral lesions compared to standard DWI by higher spatial resolution and improved signal-to-noise ratio. In addition, MRI at 3 T enables a much higher contrast-to-noise ratio, thereby improving diagnostic reliability of stroke detection, while the detection of cerebral haemorrhage, chronic lesions and brain vessel occlusion is at least equal to 1.5 T MRI [32]. Using the portable ECG-recorder CardioMem® CM 4000 we will be able to correlate the rate of brain lesions with the burden of cardiac arrhythmias. The CardioMem® CM 4000 was successfully tested by a few athletes during the 37^th^ real,- BERLIN-MARATHON 2010 [GETEMED AG, Teltow, Germany; personal communication]. So far there is only one comparable study done back in 1994. Luurila et al. monitored the ECG of 37 healthy middle-aged men during a ski marathon in Finland. They reported ST-segment elevations in a few participants during the ski marathon, an increasing number of ventricular premature complexes but no clinically relevant cardiac arrhythmias [22].

Contrast-enhanced cardiac MRI in selected patients will enable us to assess pathological changes in the heart of those participants with brain lesions or cardiac arrhythmias [33]. Moreover, (subclinical) myocardial infarction can be ruled out in those patients with troponin elevation after the marathon [34]. Serial laboratory tests will allow drawing conclusions regarding the relationship between exercise and endothelial function, dehydration, renal function or the release of brain-specific biomarkers, respectively. Performing an echocardiogram or cardiac MRI on all participants would have added valuable information to the study. However, due to time restrictions, cardiac imaging was restricted to selected participants with pathological ECG findings or significant troponin elevation during the marathon race.

The data presented here arise from the initial clinical visit immediately before the 38^th^ BMW BERLIN-MARATHON 2011. Not surprisingly, the data indicate a very low cardiovascular risk profile in study participants further suggesting a low risk for cardiac arrhythmias per se.

As depicted in Table 2, our data will allow comparisons across gender, which are missing so far [10]. Further major advantages of the “Berlin Beat of Running” study are the high technical standard, and the homogeneous cohort of experienced endurance runners without known cardiac arrhythmia. However, the “Berlin Beat of Running” study has some limitations. First, focusing on experienced marathon runners, we are unable to address questions regarding those athletes running their first marathon or those athletes who are insufficiently trained to run a marathon. Second, due to logistic reasons, the number of participants was limited. Third, ECG-recording during the marathon might be difficult to interpret because of movement artefacts. Fourth, regarding the frequency of cardiac arrhythmias in marathon runners a concurrent “non-athletic” control group might be useful.

Taken together, the “Berlin Beat of Running” study is a prospective observational study in experienced marathon runners. The results will provide further information on the effects of vigorous physical activity on heart and brain and will help to better counsel endurance athletes regarding the risks and benefits of marathon running.

## Abbreviations

AF, Atrial fibrillation; ECG, Electrocardiogram; MRI, Magnetic resonance imaging; T, Tesla.

## Competing interests

KGH reports lecture fees from Sanofi and Bayer Healthcare. MK reports receiving consulting, lecture and advisory board fees from Novartis, Takeda, MSD, GlaxoSmithKline and Janssen-Cilag. JBF reports receiving consulting, lecture and advisory board fees from BMS, Siemens, Philips, Perceptive, Bio-Imaging Technologies, Boehringer Ingelheim, Lundbeck and Sygnis. ME reports consulting, lecture, grant and/ or advisory board fees from BMS, Sanofi, Boehringer-Ingelheim, Novartis, Pfizer and AstraZeneca. GJJ received consulting and lecture fees from Bayer Health Care, Boehringer Ingelheim, Genzyme, Novartis, Pfizer, Sanofi and Takeda.

## Authors’ contributions

All authors have read and approved the final manuscript. KGH, GJJ, JH and CK initiated the study. ME, WH, JL, LB and MK added substantially to its final form. KGH, JH and GJJ wrote the study-protocol and the draft of the manuscript. The MRI examinations were primarily done by CK under supervision of JBF, who will do the blinded MRI reading in addition. Data management was primarily done by MH. PUH did and will do the statistical calculations. The assessment of cardiac arrhythmias is supervised by WH.

## Pre-publication history

The pre-publication history for this paper can be accessed here:

http://www.biomedcentral.com/1471-2261/12/69/prepub
